# Nanotribological Characteristics of the Al Content of Al_x_Ga_1−x_N Epitaxial Films

**DOI:** 10.3390/nano13212884

**Published:** 2023-10-31

**Authors:** Hua-Chiang Wen, Ssu-Kuan Wu, Cheng-Wei Liu, Jin-Ji Dai, Wu-Ching Chou

**Affiliations:** Department of Electrophysics, College of Sciences, National Yang Ming Chiao Tung University, Hsinchu 30010, Taiwan; wusykuann@gmail.com (S.-K.W.); william798424@gmail.com (C.-W.L.); jinjidai@gmail.com (J.-J.D.)

**Keywords:** metalorganic vapor phase epitaxy, friction, nanoscratch

## Abstract

The nanotribological properties of aluminum gallium nitride (Al_x_Ga_1−x_N) epitaxial films grown on low-temperature-grown GaN/AlN/Si substrates were investigated using a nanoscratch system. It was confirmed that the Al compositions played an important role, which was directly influencing the strength of the bonding forces and the shear resistance. It was verified that the measured friction coefficient (μ) values of the Al_x_Ga_1−x_N films from the Al compositions (where x = 0.065, 0.085, and 0.137) were in the range of 0.8, 0.5, and 0.3, respectively, for Fn = 2000 μN and 0.12, 0.9, and 0.7, respectively, for Fn = 4000 μN. The values of μ were found to decrease with the increases in the Al compositions. We concluded that the Al composition played an important role in the reconstruction of the crystallites, which induced the transition phenomenon of brittleness to ductility in the Al_x_Ga_1−x_N system.

## 1. Introduction

Aluminum gallium nitride (Al_x_Ga_1−x_N) is a material in III-Nitride alloys, which have an appliable tunable bandgap under changeable compositions of their elements, and they have applications for optical, optoelectronic, high-power, and sensor devices [1,2,3,4,5]. These materials are always concentrated to reduce the negative influences of strain and stress on these devices, and so it is reasonable to improve the electron mobility, working stability, and device performance, even if the problems are from crystal distortion, microcracking, and the machining process [6]. It is interesting that Al_x_Ga_1−x_N films (in MBE systems) are grown on LT-GaN/AlN/Si (111) substrates (in MOCVD systems). AlGaN as an intermediate layer has been successfully introduced between the AlN nucleation layer and the GaN to prevent the cracking of the GaN [7,8].

However, the reliability of a microelectronic device usually suffers due to issues in the processes of preparation, packaging, and transportation. Zeng et al. reported on the wear and tribochemical performances of GaN specimens, which exhibited extremely low wear rates [9], and in related studies, the behavior of the GaN was highly dependent on the crystal orientation [10] and moisture level [11]. In a recent study, issues related to the nanoindentation characterization of GaN epilayers on A-axis sapphire substrates [12] and the bundled effect of crystalline GaN nanocolumns were discussed [13]. Nanoscratch equipment can be applied as a tool to assess the adhesion of films at relatively large contact pressures. Previously, we reported that the nanotribological property is a key factor that influences the ZnMgO epilayers on an M-plane sapphire’s efficiency and lattice damage behavior [14]. For the issue of nanotribological characterizations, we applied a very sharp tip to a ploughing GaN hexagonal lattice structure, and the C axis was more shear-resistant than the A-axis of the GaN [15]. Due to variations in the internal behaviors of materials, the nanoscratch equipment as a system may provide the basis for novel studies. Tan et al. [16] reported that due to the abrasive machining process for the GaN sublayer, plastic deformations were dominated by nanoscratch slips under relatively low and normal loads, and dislocations and lattice distortions were observed using transmission electron microscopy. Guo et al. [17] reported that the mechanochemical reactions on Ga- and N-faced GaN surfaces rubbed with an Al_2_O_3_ nanoasperity were functions of the environmental humidity. The nanoscratch reactions were achieved in several of the atomic layers in the contact interface and were responsible for the mechanochemical removal behaviors of the Ga- and N-faced GaN surfaces. However, it remains challenging to investigate high-quality Al_x_Ga_1−x_N films, even generally, as it is difficult to recreate the strain–stress and reconstruction phenomena of the crystallites [18]. The use of Al_x_Ga_1−x_N films usually results in poor device performances owing to the extensive defects and serious cracking at the inter-surfaces by means of variations in the Al mole fractions [19,20]. While most of the issues are concentrated on the optoelectronic characteristics, studies on their nanotribological characterizations have not drawn equal attention. Interestingly, it has been thought that Al_x_Ga_1−x_N films exhibit advantages in obtaining nanotribological properties and atomic-level damage mechanisms by means of their changeable Al compositions.

In this study, we employed a nanoscratch indenter to slide through the surfaces of Al_x_Ga_1−x_N films under continuously lateral and constant loads. The surface morphologies and delamination distributions were analyzed using atomic force microscopy and nanoscratch methods. The critical role of Al composition can result in a well-defined slide curve of the jumping event.

## 2. Materials and Methods

The samples were prepared as Al_x_Ga_1−x_N (PA-MBE)/LT-GaN/AlN (MOCVD)/Si (111) substrates in two steps [21]. For step (I), the full structures of the GaN-based HEMTs were grown on six-inch Si (111) substrates using a G4 MOCVD system (Aixtron, Herzogenrath, Germany). Conventional precursors, including TMGa, TMAl, NH_3_, and silane (1% diluted-SiH_4_), were used for the Ga, Al, N, and Si sources, respectively. To grow the lattice-matched GaN layer on the Si (111) substrate, an AlN nucleation layer of approximately 225 nm was used. A semi-insulating LT-GaN buffer layer with a thickness of 1.43 µm was then deposited at 990 °C.

For step (II), the Al_x_Ga_1−x_N epi layers were grown on LT-GaN/AlN/Si (111) substrates in a high vacuum PA-MBE system [13]. To fabricate the Al_x_Ga_1−x_N films on the LT-GaN/AlN/Si (111) substrates, an AlN buffer layer (5 nm) was deposited as a catalyst. The molecular fluxes were calibrated on the AlN buffer layer on LT-GaN/AlN/Si (111) substrates for the Al_x_Ga_1−x_N growth rate units (1 nm/min) at a high vacuum of 4 × 10^−8^ mbar.

In addition, there was a constant Ga temperature (950 °C) while the active Al temperature was maintained. Only the growth temperatures were changed from the nitride-plasma conditions (Ga/N flux ratio of <0.4), and the radio-frequency (RF) plasma power was 800 watts and the N_2_ flow rate was 0.9 sccm. In terms of the impinging fluxes, there was a constant substrate temperature of 700 °C. The growth duration was 330 min for each of the samples at the different active Al temperatures. The pressure was kept at 9.83 × 10^−8^ mbar, and the Al effusion-cell temperatures were 940, 950, and 975 °C as the Al content was adjusted for x = 0.065, 0.085, and 0.137, respectively. 

Scanning electron microscopy (SEM) using a JEOL JSM-7001F (operated at 28 keV and 18 nA) was performed to observe the Al_x_Ga_1−x_N/sapphire system. A scanning probe microscopy (SPM) measurement system (Hysitron), operating at a constant scan speed of 2 μm s^−1^, with a nanoindenter tip was used to perform the nanoscratch tests. After the nanoscratch tests, an atomic force microscope system (AFM, Digital Instruments Nanoscope III, Hysitron Inc., Minneapolis, MN, USA) was used. Calculations were performed with the scanning probe image processor (SPIP) software, which is a standard program for processing AFM data at the nanoscale. The Al_x_Ga_1−x_N films were evaluated for both types of constant force (2000 and 4000 μN, respectively), the so-called “Fn”, and the respective maximum load was applied to form a 10-µm-long scratch. Surface profile pictures before and after scratching were captured by scanning with a tip at a normal load of 0.02 mN by applying an AFM. An atomic force microscope (AFM, Digital Instruments Nanoscope III) and a nanoindentation measurement system (Hysitron), operating at a constant scan speed of 2 μm s^−1^, were used to perform the nanoindenter tests.

## 3. Results

**Figure 1:**
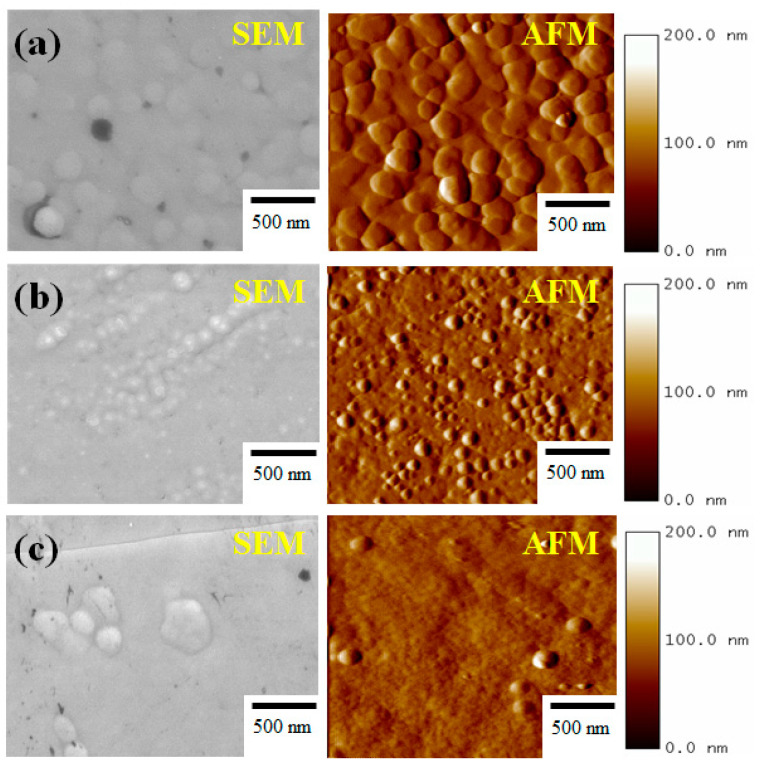
SEM/AFM images of the Al_x_Ga_1−x_N films on the substrates. The surface roughness (RMS) increased with the roughness values of 2.2, 3.2, and 7.2 nm and for the Al mole fractions of (**a**) 0.065, (**b**) 0.085, and (**c**) 0.137, respectively.

Figure 1 shows the SEM/AFM images of the Al_x_Ga_1−x_N film systems. The surface roughness (RMS) increased with the roughness values of 2.2, 3.2, and 7.2 nm for the Al mole fractions of (a) 0.065, (b) 0.085, and (c) 0.137, respectively. By monitoring the 2D-AFM morphology, the images provided strong evidence for uniform, island-like particles and grain boundaries when the value of x was 0.065. As shown in Figure 1, however, the micro- to nano-scale sizes of the grain boundaries were observed when the values of x were 0.085 and 0.137. It was agreed that the combinations of surface energies were driven by the MBE growth, and therefore, the migration of the grain boundaries tended to form island-like particles as the Al mole fractions were increased [8,13]. The effects of the Al mole fractions on the adhesive failures of the Al_x_Ga_1−x_N films were analyzed in real-time by using nanoscratch measurements. Herein, the formations of the nanoscratch traces and the scale bar distributions of the Al_x_Ga_1−x_N films were compared for the measurements of Fn = 2000 μN and 4000 μN. 

**Figure 2:**
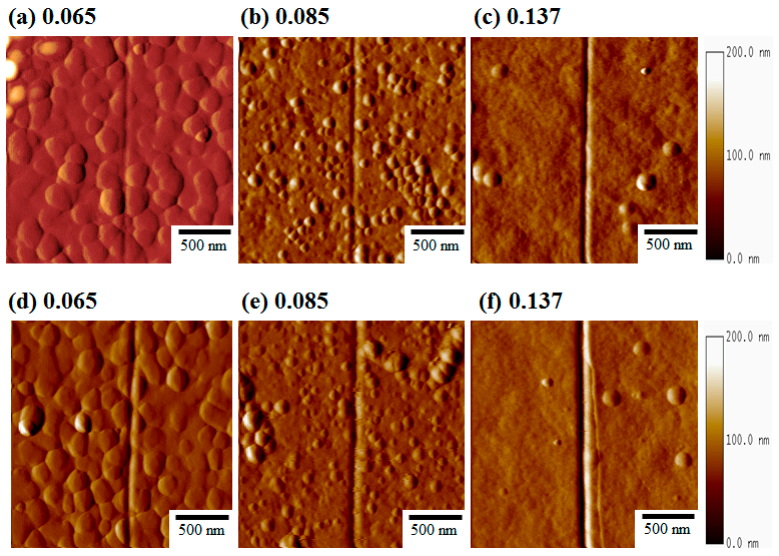
AFM images of the nanoscratch sliding: Fn = 2000 μN for the Al mole fractions of (**a**) 0.065, (**b**) 0.085, and (**c**) 0.137, respectively, and Fn = 4000 μN for the Al mole fractions of (**d**) 0.065, (**e**) 0.08, and (**f**) 0.137, respectively.

Figure 2 presents AFM images of the nanoscratch sliding where the lateral forces (Fn) varied for 2000 μN and 4000 μN, corresponding to the Al mole fractions of (a) 0.065, (b) 0.085, (c) 0.137, (d) 0.065, (e) 0.08, and (f) 0.137, respectively. Eventually, the surface damage to the parent material suffered from the nanoscratch slide, and the plastic/elastic behavior could be determined from the lateral force. The sliding area and the scratch penetration were increased depending on the increase in the Fn from 2000 μN to 4000 μN. We determined that the area of the nanoscratch trace at Fn = 2000 μN was actually smaller than that at Fn = 4000 μN (Figure 2a–c). It is believed that nonfit dislocations are relaxed at applied loads of 2000 μN between the elastic/plastic phenomenon [14,16,17]. Furthermore, the sliding tracks were observed to have some debris because of the damage from the slide track (Figure 2d–f). The plastic tracks appeared at the end of the scratch functions for Fn = 4000 μN (Figure 2f), which exhibited a larger damage area and a deeper sliding track than others (Figure 2d,e). We concluded that the proportion of the plastic deformation of the debris on the surface area gradually increased with the increase in Fn, which resulted in the observation of the transition of the brittle to ductile fractures [22]. Furthermore, the penetration depth and groove width increased as the Fn increased from 2000 μN to 4000 μN. We continued to report the load-displacement curve versus the entire scratching process and to investigate the distinct fluctuations in the nanoscratch process. 

**Figure 3:**
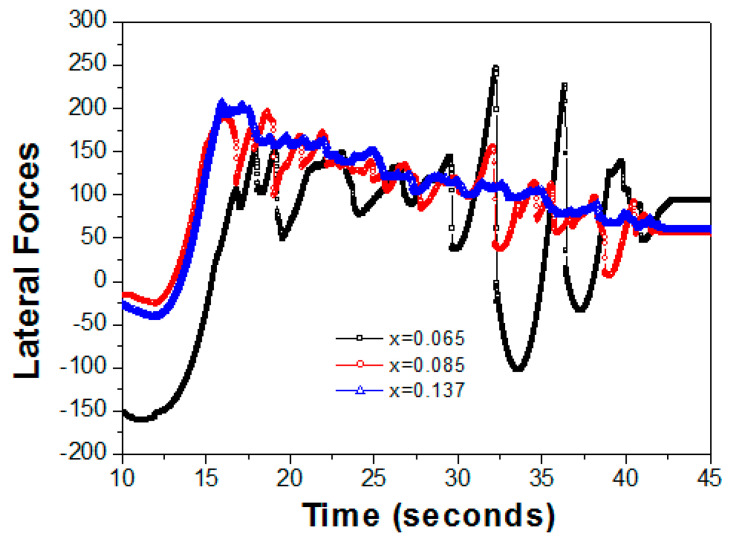
The lateral forces (2000 μN) versus the time for the three Al mole fractions of 0.065, 0.085, and 0.137.

Each nanoscratch depth could be measured using a constant Fn function. The plot of the critical load depending on the depth indicated that the film had gone under the slide track as a result of its behaviors. Figure 3 shows the asperity tips on the contacting grain boundaries for the observable slide track at Fn = 2000 μN. At the condition of x = 0.065, the high brittleness damage of the rupturing curve was dependent on the formation of the grain boundaries. The vibration range versus the lateral forces ranged from 150 μN to −50 μN and the vibrations lasted for 17 to 30 s, with another rising wave from 250 μN to −100 μN, which lasted for 30 to 40 s. 

The low brittleness damage from the rupturing curve was observed at x = 0.085, and the vibration range versus the lateral forces was from 10 to 180 μN at the total length of time. A slightly machined area for the constant force was observed at Fn = 2000 μN. One could separate the sliding surface and the brittleness damage, which produced delaminated craters on the worn surface as the transition of the Al mole fractions went from x = 0.065 to x = 0.085. The shearing curve and adhesive surface, as results of the local contact pressure during sliding, were obtained, as shown in the AFM images in Figure 2a,b. However, this was attributed to the ductility fracture increasing dramatically, and the curved cracks aligned roughly perpendicular to the direction of the nanoscratching that was observed at x = 0.137 (Figure 3), as shown in AFM image in Figure 2c. This failure event represents the detachment of the curve from the vibration range versus the lateral forces, and the critical loads of the rupturing curve could be used as a qualitative adhesion measurement of the epitaxial orientation on the Al_x_Ga_1−x_N films [14]. 

**Figure 4:**
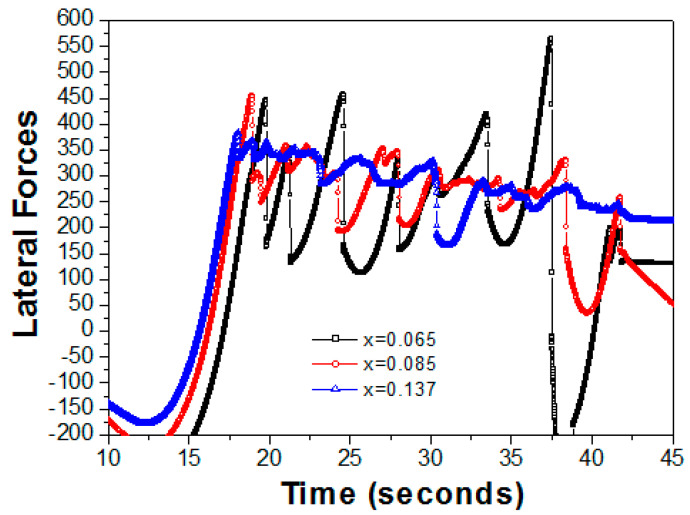
The lateral forces (4000 μN) versus time for the three Al mole fractions of 0.065, 0.085, and 0.137.

Figure 4 shows the three conditions of the lateral forces versus the nanoscratch duration, and these fluctuations in the specific lateral values for Fn = 4000 μN were observed as well. The high brittleness damage from the rupturing curve was observed at x = 0.065, and the vibration of the curve depended on the formation of the grain boundaries [16,17]. The vibration range versus the lateral forces were from 100 μN to 420 μN and the duration was 17–30 s. Another rising wave versus the lateral forces was observed from 580 μN to −250 μN and it lasted for 30–40 s. The low brittleness damage from the rupturing curve was observed at x = 0.085, and the average vibrations versus the lateral forces ranged from 200 μN to 400 μN for a duration of 25–40 s. The ductility fracture was observed at x = 0.137, and the lateral forces increased gradually with stable vibrations. There was a rising wave versus the lateral forces from 200 to 350 μN, with a duration of 25–40 s. From the observation of the continued duration, the regularities appeared in the course of the plastic deformation and depended on the effects of the adhesion and/or cohesion failures [15]. The above results corresponded with the observations in the AFM images (Figure 2d–f) with the slight sliding track, and no obvious changes in the volumes of the bulge edges were observed at Fn = 2000 μN. However, the clearly visible cross-slip defects and bulge edges were clearly present at Fn = 4000 μN. Ductility damage from the rupturing curve was observed (x = 0.137) at both the lateral Fn = 2000 μN and 4000 μN values. Evidence for the direct formation of slide nanoscratches appeared in the rupturing curves. This confirmed the transition of the elastoplastic contact from pure plastic to pure elastic from the constant force of the nanoscratch tip [14,15].

**Figure 5:**
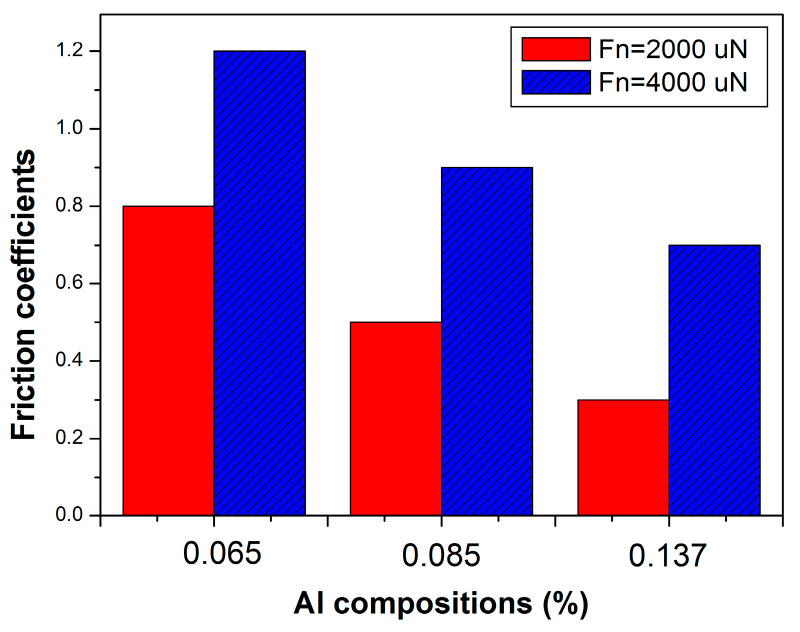
The values of μ (0.8, 0.5, and 0.3) for Fn = 2000 μN and the values of μ (0.12, 0.9, and 0.7) for Fn = 4000 μN for the Al mole fractions of 0.065, 0.08, and 0.137, respectively.

Meanwhile, the corresponding frictional coefficient (μ) values varied when Fn = 2000 μN and 4000 μN (Figure 5). When the evaluated Al mole fractions were 0.065, 0.08, and 0.137, respectively, the corresponding μ values were 0.8, 0.5, and 0.3 when Fn was 2000 μN, and they were 0.12, 0.9, and 0.7, respectively when the value of Fn was 4000 μN. It is worthwhile to point out that the value of μ decreased with the increasing Al mole fractions. The ductility damage from the rupturing curves may have been caused by the high-Al composition films (x = 0.137), and the brittleness damage occurred when x = 0.065. This phenomenon was previously discussed as being due to the tight bonds in the Ga-N and/or the Al-N, which arose from the various nanoscratch-induced deformations that were associated with the bonding bridges accessed as regular oscillations [22]. Thus, the effect of the nanotip presence on the μ values could be explained as follows: the agglomeration of the crystallites of the high-quality Al_x_Ga_1−x_N system for the high-Al composition (x = 0.137) was more pronounced than that of the low-Al composition (x = 0.065). The influence of the strength of the Al-N as well as the Ga-N bonds on the kinetics of growth of the nitride alloys was determined by the nitrogen-rich and group-III-rich growth regimes; however, the unity of the incorporation the Al atomic played an important role [6]. We concluded that the high-Al composition could have induced a reconstruction phenomenon in the crystallites, which led to the transition phenomenon of brittleness to ductility in the Al_x_Ga_1−x_N system. Drygas et al. reported that the formation of pure Al_0.5_Ga_0.5_N nanoceramics was associated with a closed pore evolution, and it had a detrimental effect on the hardness [23,24]. It was determined that the hardness was related to the film quality. For another view-point, researchers have also reported that in Al_x_Ga_1−x_N films grown by MOCVD, the variations in hardness and moduli were comparable [25,26]. It was evidenced that the alloy hardening of the Al_x_Ga_1−x_N films was confirmed, and it was attributed to the residual in-plane strain and threading dislocation densities [27]. In contrast, from the MBE growth of the Al_x_Ga_1−x_N films, the incorporation probability of the Ga decreased monotonically with the total group-III flux, which was observable in the competition with the Al for the available active nitrogen. The SEM/AFM surface morphologies were island-like, and the RMS values were strongly affected by the crystal qualities [24]. One cause of dislocation was attributed to the few transitions from the inner films, presumably from the Al mole fractions when x was 0.137, which involved a high-level point defect or interstitial defects with the Al_x_Ga_1−x_N films. Defects are known to exist throughout Al_x_Ga_1−x_N films at the critical Al mole fractions ranging from 0.065 to 0.137, and hence, they could lead to dislocation trapping. Cherns et al. [28] discussed how screw dislocations act as strong nonradiative centers, and even edge dislocations do not act as nonradiative centers. Arslan and Browning [29] studied the resultant electrical activity at dislocations due to the segregation of dopants, impurities, and vacancies. 

The μ values versus constant loads for Fn = 2000 μN and Fn - 4000 μN were comparable for the Al compositions. At Fn = 2000 μN, an integrated Al mole fraction was formed near the sliding surface, which provided the real contact area. With increasing Al mole fractions, the variations in the extent of the value of μ for Fn = 2000 μN was lower than that for Fn = 4000 μN. We ensured that the real contact area and the frictional force applied to the films were the major factors affecting the μ values (Figure 5). The μ values were dependent on the values of Fn (2000 and 4000 N), which decreased as a function of the increased Al mole fraction. First, the μ value was 0.3 at x = 0.137, which was small compared with the μ values of 0.5 and 0.8 (for x = 0.065 and 0.085) due to the tight bonds relaxing between the Al-N and/or the Ga-N bonds and the crystal. Second, when Fn = 4000 μN, it resulted in more oscillating fluctuations than when Fn = 2000 μN because the nanoscratch-induced tight bonds suddenly relaxed from the nanotip. Third, the resultant Al composition films were formed at x = 0.137; however, they exhibited the lowest μ values. This aligned with the results for Al_x_Ga_1−x_N films where the hardness and Young’s moduli were measured by a Berkovich nanoindenter operated in the continuous contact stiffness measurements mode. The Al compositions were estimated using nanoindentation and ranged from x = 0.065 to 0.136, and the H values ranged from 18.5 ± 0.2 to 19.2 ± 1.1 GPa while the E values ranged from 125 ± 4.3 to 224 ± 1.4 GPa, respectively. At the same time, the hardness properties could be compared with the slight decreases in H values ranging from 20.26 to 19.56 GPa for Al contents ranging from 0.09 to 0.27 [30,31,32]. Nevertheless, we observed that the μ values decreased with the increases in the Al composition during the tribological test, especially for Fn = 2000 μN. Because of the phenomenon of the slide track and the surface dangling-bond configuration from the aluminum gallium nitride alloy composition, an Al-rich bond may have existed along the c-plane wurtzite GaN, and the Ga-faced GaN and N-faced GaN could be divided easily. We believed that the surface chemical bonds of the Ga- and N-faced GaN materials were involved, and the Ga-face and/or Al-face was more chemically inert than the N-face in the MBE growth. This is the reason why the higher contents of Al showed lower friction coefficient values [17]. The nanoscratch mechanism of the nanotip was demonstrated on the Al_x_Ga_1−x_N films, and the different μ values were expected because of the increased Al compositions. At the different Fn values, the effects of the visible cross-slip defects on the curves of the μ values were expected [14,15,16,17,18].

## 4. Conclusions

The investigation was focused on the nanotribological characteristics and elastic and plastic deformation behaviors of Al_x_Ga_1−x_N films with different Al compositions. We employed nanoscratch techniques to observe the morphological responses of the films. The values roughness increased slightly from 2.2 to 7.2 nm as the Al compositions increased from 0.065 to 0.137. The μ values were 0.8, 0.5, and 0.3 for Fn = 2000 μN and the μ values were 0.12, 0.9, and 0.7 for Fn = 4000 μN when the Al mole fractions were 0.065, 0.085, and 0.137, respectively. The nanoscratch mechanisms produced sudden bursts of displacement jumping curves for the lateral forces versus the durations, and the different nanoscratch periods were obtained. The Al_x_Ga_1−x_N films were dense, with larger Ga-N bonds which adhered to the brittleness at x = 0.065. However, there was greater ductility in the rich Al-N bonds at x = 0.137. This was the reason for the effects of adhesion and/or cohesion failure in the nanotribological experiments using the samples. It is obviously the friction coefficients at the different sliding forces and the different Al mole fractions that have been reported. There was a mutual relationship between the value of μ and the real contact areas at the evaluated growth conditions. These findings could be used to achieve an understanding of the nanoscratch mechanisms in the interfaces of Al_x_Ga_1−x_N films grown on LT-GaN/AlN/Si (111) substrates in a high vacuum PA-MBE system. The outcomes of the study suggest, for example, that a quantitative analysis of high-Al compositions using a ω-2θ HRXRD scan can be used to investigate wear measurements on Al_x_Ga_1−x_N films.

## Data Availability

This manuscript is the authors’ original work and has not been published nor has it been submitted simultaneously elsewhere. All authors are requested to make sure that all data and materials as well as software application or custom code support their published claims and comply with field standards.

## References

[1] Jain S.C., Willander M., Narayan J., Van Overstraeten R. (2000). III–nitrides: Growth, characterization, and properties. J. Appl. Phys..

[2] Ponce F.A. (1997). Defects and Interfaces in GaN Epitaxy. MRS Bull..

[3] Perry W.G., Bremser M.B., Davis R.F. (1998). Cathodoluminescence studies of the deep level emission bands of Al*_x_*Ga_1−x_N films deposited on 6H–SiC(0001). J. Appl. Phys..

[4] Li D., Jiang K., Sun X., Guo C. (2018). AlGaN photonics: Recent advances in materials and ultraviolet devices. Adv. Opt. Photonics.

[5] Jung S., Baik K.H., Ren F., Pearton S.J., Jang S. (2018). AlGaN/GaN Heterostructure Based Schottky Diode Sensors with ZnO Nanorods for Environmental Ammonia Monitoring Applications. ECS J. Solid State Sci. Technol..

[6] Van Nostrand J.E., Hengehold R.L., Leedy K.D., Grant J.T., Brown J.L., Xie Q.-H. (1999). Cathodoluminescence, microstructure, and morphology of tensile-strained Al*_x_*Ga_(1−x)_N epitaxial films grown by gas source molecular beam epitaxy. J. Appl. Phys..

[7] Able A., Wegscheider W., Engl K., Zweck J. (2005). Growth of crack-free GaN on Si(111) with graded AlGaN buffer layers. J. Cryst. Growth.

[8] Cheng K., Leys M., Degroote S., Van Daele B., Boeykens S., Derluyn J., Germain M., Tendelloo V., Engelen J., Borghs G. (2006). Metalorganic Vapor Phase Epitaxy Using Step-Graded AlGaN Intermediate Layers. J. Electron. Mater..

[9] Zeng G., Tan C.K., Tansu N., Krick B.A. (2016). Ultralow wear gallium nitride. Appl. Phys. Lett..

[10] Zeng G., Sun W., Song R., Tansu N., Krick B.A. (2017). Crystal orientation dependence of gallium nitride wear. Sci. Rep..

[11] Zeng G., Tansu N., Krick B.A. (2018). Moisture dependent wear mechanisms of gallium nitride. Tribol. Int..

[12] Lin M.H., Wen H.C., Chang Z.C., Wu S.C., Wu W.F., Cho C.P. (2010). Effect of Repetition Nanoindentation of GaN Epilayers on a-axis Sapphire Substrates. Surf. Interface Anal..

[13] Wen H.C., Chou W.C., Chiang T.Y., Jeng Y.R., Fan W.C. (2017). Using nanoindentation and cathodoluminescence to identify the bundled effect of gallium nitride grown by PA-MBE. J. Alloys Compd..

[14] Wen H.C., Chou W.C., Chiang T.Y., Fan W.C., Lee L. (2015). Scratch Characteristics of ZnMgO Epilayers. Tribol. Lett..

[15] Lin M.H., Wen H.C., Jeng Y.R., Chou C.P. (2010). Nanoscratch characterization of GaN epilayers on c-and a-axis sapphire substrates. Nanoscale Res. Lett..

[16] Tan S., Wang Y., Huang H., Wu Y., Huang H. (2022). Deformation and removal mechanism of single crystal gallium nitride in nanoscratching. Ceram. Int..

[17] Guo J., Gao J., Xiao C., Chen L., Qian L. (2022). Mechanochemical reactions of GaN–Al_2_O_3_ interface at the nanoasperity contact: Roles of crystallographic polarity and ambient humidity. Friction.

[18] Reisinger M., Tomberger M., Zechner J., Daumiller I., Sartory B., Ecker W., Keckes J., Lechner R.T. (2017). Resolving alternating stress gradients and dislocation densities across Al_x_Ga_1−x_N multilayer structures on Si(111). Appl. Phys. Lett..

[19] Feng Y., Saravade V., Chung T.-F., Dong Y., Zhou H., Kucukgok B., Ferguson I.T., Lu N. (2019). Strain-stress study of Al*_x_*Ga_1−*x*_N/AlN heterostructures on c-plane sapphire and related optical properties. Sci. Rep..

[20] Xu Q., Liu B., Zhang S., Tao T., Dai J., He G., Xie Z., Xiu X., Chen D., Chen P. (2017). Superlattices and Microstructures Structural and optical properties of Al_x_Ga_1−x_N (0.33 ≤ x ≤ 0.79) layers on high-temperature AlN interlayer grown by metal organic chemical vapor deposition. Superlattices Microstruct..

[21] Dai J.J., Liu C.W., Wu S.K., Huynh S.H., Jiang J.G., Yen S.A., Mai T.T., Wen H.C., Chou W.C., Hu C.W. (2021). Improving Transport Properties of GaN-Based HEMT on Si (111) by Controlling SiH_4_ Flow Rate of the SiN_x_ Nano-Mask. Coatings.

[22] Guo J., Qiu C., Zhu H., Wang Y. (2019). Nanotribological Properties of Ga- and N-Faced Bulk Gallium Nitride Surfaces Determined by Nanoscratch Experiments. Materials.

[23] Drygas M., Kapusta K., Janik J.F., Bucko M.M., Gierlotka S., Stelmakh S., Palosz B., Olejniczak Z. (2020). Novel nanoceramics from in situ made nanocrystalline powders of pure nitrides and their composites in the system aluminum nitride AlN/gallium nitride GaN/aluminum gallium nitride Al_0.5_Ga_0.5_N. J. Eur. Ceram..

[24] Jian S.R., Juang J.Y., Lai Y.S. (2008). Cross-sectional transmission electron microscopy observations of structural damage in Al_0.16_Ga_0.84_N thin film under contact loading. J. Appl. Phys..

[25] Jian S.R., Fang T.-H., Chuu D.-S. (2003). Analysis of Physical Properties of III-Nitride Thin Films by Nanoindentation. J. Electron. Mater..

[26] Boughrara N., Benzarti Z., Khalfallah A., Oliveira J.C., Evaristo M., Cavaleiro A. (2022). Thickness-dependent physical and nanomechanical properties of thin films. Mater. Sci. Semicond. Process..

[27] Li C., Piao Y., Meng B., Hu Y., Li L., Zhang F. (2022). Phase transition and plastic deformation mechanisms induced by self-rotating grinding of GaN single crystals. Int. J. Mach. Tools Manuf..

[28] Monroy E., Daudin B., Bellet-Amalric E., Gogneau N., Jalabert D., Enjalbert F., Brault J., Barjon J., Dang L.S. (2003). Surfactant effect of In for AlGaN growth by plasma-assisted molecular beam epitaxy. J. Appl. Phys..

[29] Cherns D., Henle S.J., Ponce F.A. (2001). Edge and screw dislocations as nonradiative centers in InGaN/GaN quantum well luminescence. Appl. Phys. Lett..

[30] Spikes H. (2015). Friction Modifier Additives. Tribol. Lett..

[31] Touré A., Halidou I., Benzarti Z., Fouzri A., Bchetnia A., El Jan B. (2012). Characterization of low Al content Al*_x_*Ga_1−*x*_N epitaxial films grown by atmospheric-pressure MOVPE. Phys. Status Solidi.

[32] Jayasakthi M., Ramesh R., Loganathan R., Prabakaran K., Balaji M., Baskar K. (2014). Structural and optical characterization of AlGaN/GaN layers. J. Cryst. Growth.

